# Poly(l-lactic acid)-based double-layer composite scaffold for bone tissue repair

**DOI:** 10.1093/rb/rbad093

**Published:** 2023-10-27

**Authors:** Yixing Ren, Chunyang Ma, Yao Yu, Dandan Yang, Lingling Zhang, Huitao Wang, Lei Sun

**Affiliations:** Department of Joint Surgery, The Fourth Central Hospital of Baoding City, Baoding 072350, China; School of Biological Science and Medical Engineering, Beihang University, Beijing 100083, China; School of Biological Science and Medical Engineering, Beihang University, Beijing 100083, China; Department of Science and Education, The Fourth Central Hospital of Baoding City, Baoding 072350, China; Department of Nursing, The Fourth Central Hospital of Baoding City, Baoding 072350, China; Department of General Surgery, The Fourth Central Hospital of Baoding City, Baoding 072350, China; School of Biological Science and Medical Engineering, Beihang University, Beijing 100083, China; School of Engineering Medicine, Beihang University, Beijing 100083, China

**Keywords:** polylactic acid, magnesium, double-layer scaffolds, controlled release, bone repair

## Abstract

Bone defect is a serious threat to human health. Osteopractic total flavone (OTF) extracted from Rhizoma Drynariae has the effects of promoting bone formation. Panax notoginseng saponin (PNS) has the function of activating blood circulation and removing blood stasis. Therefore, combining OTF and PNS with poly(l-lactic acid) (PLLA) to prepare scaffolds containing PNS in the outer layer and OTF in the inner layer is a feasible solution to rapidly remove blood stasis and continue to promote bone formation. In addition, degradation rate of the scaffold can affect the release time of two drugs. Adding Mg particles in outer layer can control the degradation rate of the scaffold and the drug release. Therefore, a double-layer drug-loaded PLLA scaffold containing OTF in the inner layer, PNS and Mg particles in the outer layer was prepared and characterized to verify its feasibility. The experimental results showed that the scaffold can realize the rapid release of PNS and the continuous release of OTF. With the increase of Mg content, the drug release rate became faster. Animal experiments showed that the scaffold containing 5% Mg particles could effectively promote the formation of new bone in the bone defect of male New Zealand white rabbits, and the area and density of new bone formed were much better than those in the control group. These results demonstrated that the double-layer drug-loaded scaffold had good ability to promote bone repair.

## Graphical Abstract

**Figure:**
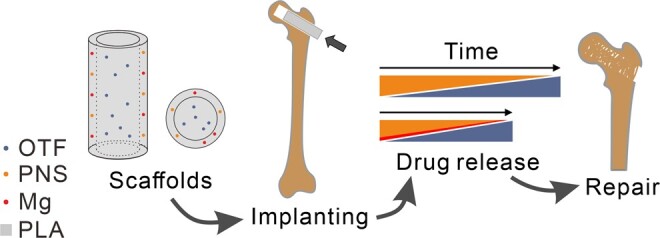

## Introduction

Bone has always been an indispensable role for the human body, not only to provide support for human activities but also to protect internal organs from impact [1]. However, large bone defects seriously affect the life activities of patients, and it is difficult to achieve self-healing [2–6]. Autogenous bone transplantation can effectively promote the repair of the defect, but it is limited by the problems of secondary injury to patients [7, 8]. The strategy of allogeneic bone transplantation is faced with the problems of immune rejection and inducing infectious diseases [7, 9]. Tissue engineering provides a new idea to solve this problem, that is, through a series of engineering designs, using biomaterials with good biocompatibility and degradability, to prepare scaffolds [10–15]. After being implanted into the human body, the scaffold plays a role in regulating the physiological microenvironment of the defect site, thereby promoting bone regeneration. With the development of research, multifunctional composite scaffold has shown great potential in the field of bone tissue repair in recent years [16, 17].

Osteopractic total flavone (OTF) has the effect of resisting osteoporosis, improving bone density and inducing osteogenesis of cells by increasing the activity of alkaline phosphatase, the level of osteocalcin, the expression of type I collagen, osteocalcin and osteopontin mRNA [18, 19]. It is an ideal drug for bone repair. However, the rupture of blood vessels in the defect area will lead to the formation of local hematoma and hinder bone regeneration. Large hematoma will not be easily absorbed by human body, or even cause infection, which requires another operation. Panax notoginseng saponin (PNS) is an effective pharmaceutical ingredient extracted from Panax notoginseng, which can improve blood rheology by reducing blood viscosity, accelerate the blood supply of fracture site, improve microcirculation and thus accelerate the healing of defect site. However, OTF and PNS cannot be directly used for the preparation of scaffolds, so it is a good choice to choose a polymer material with good biocompatibility and easy to prepare scaffolds. Poly(l-lactic acid) (PLLA) is a biocompatible biomaterial, and its degradation products have no toxic effect on human body [20–22]. However, the simple blending of OTF and PNS in PLLA scaffolds cannot effectively exert the combined effects of the two drugs, and PNS should be firstly allowed to rapidly release to drive out the hematoma, promote blood circulation, and then continuously release OTF to promote osteogenesis. Double-layered composite scaffolds were prepared with PLLA/OTF (PO) composite as the inner core and PLLA/PNS (PP) composite as the outer shell, which are expected to achieve the purpose of first removing hematoma and then promoting osteogenesis.

Common scaffold preparation methods are diverse, including 3D printing, freeze-drying, phase separation, electrospinning etc. [23–26]. In the field of bone repair, the scaffold with a coherent pore structure is more conducive to the growth of cells and tissues to promote bone regeneration [27]. Phase separation is a common method to prepare 3D scaffolds. Among them thermally induced phase separation technique has good controllability over scaffold structure including porosity, pore size and pore interconnectivity. At the same time, PLLA scaffold prepared by phase separation method can not only meet the requirements of the scaffold for through porosity, but also retain certain mechanical properties for the scaffold. The diameter of electrospun fibers is usually in the range of nanometers to submicrons, with large specific surface area and high porosity, which is conducive to cell attachment and proliferation. Moreover, the larger surface area of electrospun fibers can enhance the permeability of oxygen and accumulated fluid, thereby promoting wound healing. Nanofibers can also simulate the structure and morphology of extracellular matrix, act as mechanical support and regulator to improve cell activity before host cells refill and synthesize new matrix [28, 29]. Therefore, using the thermally induced phase separation method to prepare PO core, using the electrospinning method to prepare PP membrane, and using the PP membrane to wrap the PO core, can prepare a double-layer drug-loaded scaffold for bone defect repair.

The degradation rate of outer layer will affect the overall degradation rate of scaffold and the timing of the release of the two drugs. So the degradation rate of outer layer needs to be controlled. The incorporation of Mg nanoparticles into PLLA can not only regulate the degradation rate of PLLA, but also neutralize the acidic products produced by PLLA degradation, enhance the viability of mesenchymal stem cells, promote the macrophage mediated regulation of inflammatory response to degradation products, thus promoting bone integration and reducing host inflammatory response [30, 31]. In addition, Mg is also an important metal element in human body. Mg^2+^ is the fourth largest cation in human body, accounting for 0.72 wt% of bone. Mg deficiency will have adverse effects on all stages of bone metabolism, resulting in bone growth being stopped, osteoblast and osteoclast activity decreasing, bone loss and fragility. Therefore, the incorporation of Mg particles into outer layer of scaffold is not only expected to control the degradation rate, but also to balance pH, enhance the activity of osteoblasts and osteoclasts, and promote osteogenesis.

Therefore, a double-layer drug-loaded scaffold with PP electrospun membrane as shell and PO thermally induced phase separation porous 3D structure as core was prepared in this article, and it was characterized by multiple tests. The application prospects of this scaffold in bone tissue engineering have been explored, providing new ideas for the preparation of bone tissue repair scaffolds.

## Materials and methods

### Preparation of scaffolds

#### Purification of PLLA

Dissolve PLLA (Evonik Industries AG, Germany) particles in chloroform (Beijing Chemical Reagent Company, China) solution and stir fully at room temperature until completely dissolved. Then slowly pour the dissolved PLLA into anhydrous methanol (Beijing Chemical Reagent Company) and use glass rod to stir continuously to collect purified PLLA. After the anhydrous methanol becomes turbid, recover the waste liquid and add clean anhydrous methanol again until the purified PLLA is completely collected. Put the recovered PLLA into an electric blast drying oven (DGG-9240B, Shanghai Senxin Experimental Instrument Co., Ltd, China), dry it at 37°C for 3 days and store it for future use.

#### Preparation of porous PLLA scaffold by phase separation method

Dissolve PLLA in 1,4-dioxane (Shanghai McLean Biochemical Technology Co., Ltd, China) to a concentration of 10 wt%, stir and dissolve it in a 60°C thermostatic water bath (Feb-85, Shanghai Sile Instrument Co., Ltd, China). Add deionized water (from CASCADA MK2, Pall Company, USA) to make 1,4-dioxane:deionized water = 87:13, continue to stir at constant temperature to homogeneous transparent solution, then transfer the solution to polytetrafluoroethylene mold (customized, China), put it back into the water bath, heat induced phase separation and gel for a period of time, and then put it into −80°C refrigerator (BC/BD-379HB, Haier Group, China) for freezing. Then it is put into a freeze dryer (7960071, LABCONCO, USA) to freeze dry to obtain porous PLLA scaffolds.

#### Preparation of porous PO scaffold by phase separation method

Dissolve PLLA in 1,4-dioxane to a concentration of 10 wt%, and stir and dissolve it in a constant temperature water bath at 60°C; add deionized water to make 1,4-dioxane:deionized water = 87:13, continue to stir at constant temperature to homogeneous transparent solution, then add OTF (self-made, China) with a concentration of 10%, stir evenly, and the other steps are the same as ‘Preparation of porous PLLA scaffold by phase separation method’ to obtain drug-loaded porous PO scaffold.

#### Preparation of PLLA/PNS/Mg film by electrospinning

The mixed solution of tetrahydrofuran (THF, Shanghai McLean Biochemical Technology Co., Ltd, China)/N,N-dimethylformamide (N,N-dimethylformamide, DMF, Shanghai McLean Biochemical Technology Co., Ltd, China) with a preparation ratio of 3:1 was used as the electrospinning solution. Dissolve PLLA in THF/DMF mixed solution with a concentration of 10 wt%, heat and stir in a constant temperature water bath at 60°C to obtain a transparent and uniform PLLA spinning solution after dissolution. Then add a certain amount of PNS (self-made, China), fully stir to obtain pale yellow PP electrospinning solution. Add Mg particles of different proportions (Tangshan Weihao Magnesium Powder Co., Ltd, China) into the electrospun solution, mix evenly and turn it into gray black PLLA/PNS/Mg (PPM) electrospun solution. Use a 10 ml syringe to absorb a certain amount of electrospinning solution, then fix the syringe to the propulsion pump of the electrospinning machine (YFSP-T, Tianjin Yunfan Technology Co., Ltd, China), connect the positive pole of the power supply, use a tin foil plate to connect the negative pole of the power supply as the receiving end, adjust the voltage of the high-voltage power supply to +13 kV, −2 kV, the spinning speed to 0.0025 mm/s, and the receiving distance to 20 cm.

#### Preparation method of double-layer drug-loaded scaffold

**Figure 1.**
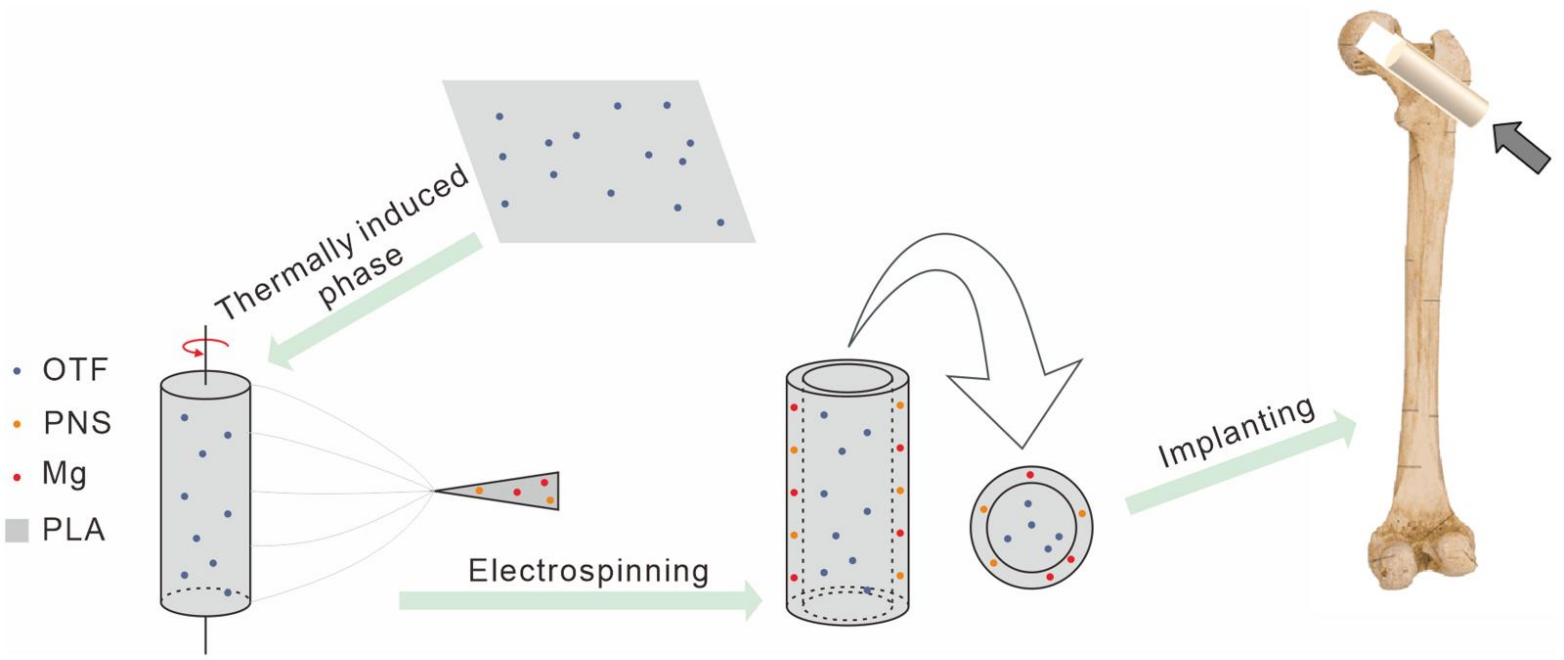
Schematic diagram of preparation method of double-layer drug carrier and scaffold implantation.

The preparation method of double-layer drug-loaded scaffold is shown in Figure 1. The inner layer porous drug-loaded PO scaffold and PPM electrospun solution were prepared according to the preparation methods in ‘Preparation of porous PO scaffold by phase separation method’ and ‘Preparation of PLLA/PNS/Mg film by electrospinning’. Place PO scaffold on the rotating motor, adjust the speed to 10 rpm, connect the negative pole of the high-voltage electrostatic spinning machine power supply as the receiving device of electrospinning, and keep the other conditions unchanged to prepare a double-layer drug carrier PLLA/OTF-PLLA/PNS/Mg (PO-PPMx, where x represents Mg content). Keep the quality of electrospun membrane on the outer layer of the scaffold in each group consistent, which is 5 mg.

### Scanning electron microscope observation method

After the prepared PLLA and PO scaffolds were quenched by liquid nitrogen, they were cut into 1 mm thick pieces and glued onto the sample stage with conductive glue before gold spraying. Spray gold two times. The gold sprayed samples were placed in a scanning electron microscope (SEM) (QUANTA 250 FEG, FEI company, the Netherlands), evacuated, and the height of the sample stage was adjusted after internal stabilization of the instrument to observe the microtopography of the samples.

The fabricated PPM electrospun membranes were tailored to 5 × 5 mm^2^ pieces and glued onto the sample stage with conductive gel and sprayed gold two times. The gold sprayed samples were put into the SEM, vacuumed and the height of the sample stage was adjusted after the internal stabilization of the instrument to observe the sample microtopography.

Put the PO-PPM0, PO-PPM5 and PO-PPM10 scaffolds into 5 ml test tubes, respectively, add 2 ml PBS buffer (Beijing Solebar Technology Co., Ltd, China), and shake at 37°C on the shaker (THZ-100, Shanghai Yiheng Instrument Co., Ltd, China) at 100 rpm. The scaffolds were collected at 12 weeks, washed with distilled water and freeze-dried. SEM was used to observe the micro morphology of the degraded scaffold.

### Aperture statistical method

The diameter of wells in scaffold sectional SEM images were measured using Image J software, counted and analyzed, counting at least of 300 wells per sample.

### Porosity test and calculation method

Use the specific gravity method to measure the porosity of the scaffolds: under constant temperature, fill the pycnometer with absolute ethanol, and measure its mass as *m*_1_. Immerse the scaffold with a mass of *m*_s_ into ethanol, vacuum and degas, fill the pores of the scaffold with ethanol, and then add ethanol. The total mass measured is *m*_2_. After taking out the scaffold filled with ethanol, the remaining ethanol and the pycnometer are weighed as *m*_3_. Calculate the porosity of the scaffold ε for:

$$\varepsilon =\frac{m_{2}\;-\;m_{3}\;-\;m_{s}}{m_{1}\;-\;m_{3}}\times 100\%.$$

### Mechanical strength test and calculation method

Use vernier caliper (DL90150, Deli Group Co., Ltd, China) to measure the diameter of the load-bearing surface of the scaffold, and then fix the scaffold on the sample platform. Open the software and enter the diameter of the scaffold. Use a universal testing machine (EZ-LX, SHIMADZU Company, Japan) to conduct a compression test to test the mechanical properties of the scaffold, and at least three parallel samples in each group. At the end of the test, the software will display the maximum compressive strength of the scaffold.

### Infrared spectrum test method

First cut the dried PP, PO and PLLA samples and put them in a clean and dry agate mortar, then add potassium bromide powder with a mass ratio of 1:100 to mix them evenly and grind them fully. Prepare the tablet pressing mould and spread the ground powder evenly on the mould. After the mold is placed under the tablet press for tablet pressing, the sample and potassium bromide are pressed into circular slices. Then put the slices into an infrared spectrometer (FTIR-7600, lambda scientific, Germany) for scanning, and the scanning wave number range is 400–4000 cm^−1^.

### Drug release and pH change curve

#### Release curve of OTF and PNS

Three samples per group of PO-PPM0, PO-PPM5 and PO-PPM10 scaffolds were placed in 5 ml tubes with 2 ml of PBS buffer, shaken at 100 rpm on a shaker at 37°C and the samples were taken at the 1, 2, 3, 4, 5, 6 and 7 days, and 1 ml of each supernatant was removed and supplemented with PBS buffer. The 0.2 ml of the supernatant was added to a quartz microplate (Shanghai Jing’An Biotechnology Co., Ltd, China), and the release of OTF was detected by UV spectrophotometry (microplate reader, multiskan FC, Thermo Co., USA). The 0.5 ml of the supernatant was taken and put into an electrothermal blast (dgg-9240b, Shanghai senxin Experimental Instrument Co., Ltd, China) drying oven at 60°C overnight for drying, followed by vanillin (Tianjin Guangfu Fine Chemical Research Institute, China)—perchloric acid (Shandong EISA Technology Co., Ltd, China) chromogenic detection of PNS release. The release curves of OTF and PNS were calculated and plotted.

#### Mg^2+^ release curve

Three samples per group of PO-PPM5 and PO-PPM10 scaffolds were placed in 5 ml tubes, added 2 ml PBS buffer, shaken at 100 rpm in a shaker at 37°C and sampled at 1, 2, 3, 4, 5, 6 and 7 days, respectively. For each experiment, 1 ml of the supernatant was removed and supplemented with 1 ml PBS buffer. The supernatant was diluted 30 times with 0.2 M HCl solution, ensuring that all Mg was dissolved, and the Mg^2+^ concentration in the supernatant was tested by using inductively coupled plasma optical emission spectrometry (inductively coupled plasma optical emission spectrometer, optima 5300DV, Perkin Elmer instruments Ltd, USA).

#### pH change curve

Three scaffolds for each group (PO-PPM0, PO-PPM5 and PO-PPM10) were placed in 5 ml tubes with 2 ml PBS buffer, and all solutions were collected at 1, 2, 3, 4, 5, 6 and 7 days by shaking at 100 rpm in a shaker at 37°C, and supplemented with 2 ml PBS solution. An acid–base meter (UB-7, Denver Instrument Company, USA) was used to measure the pH value of the solution, and the pH change curves were recorded.

### Animal experiments

#### Grouping of animals

Six adult male New Zealand large white rabbits were randomly divided into two groups of six each, and the experimental group was implanted with PO-PPM5 scaffolds, the control group was the blank defect group, and no scaffolds were implanted. The selected observation time point was 12 weeks postoperatively.

This experiment has been reviewed and approved by the ethics committee of biology and medicine of Beihang University, approval number: BM20220026.

#### Establishment of rabbit steroid-induced femoral necrosis model

New Zealand white rabbits were raised in separate cages and fed normally. On the first day, lipopolysaccharide was injected through the ear vein with the injection volume of 10 μg/kg. On the second to fourth days, the glucocorticoid methylprednisolone was injected via the gluteus medius muscle at an injection volume of 20 mg/kg, while the rabbits were provided free access to drinking water after shaking with 0.025 ml of diquali per 500 ml of water. And from the next day, until the end of modeling, penicillin was administered intramuscularly daily at 40 000 units per day. Biofermin were administered at a dose of three pills per day with a minimum interval of 6 h between the feeding and the penicillin injection. And every other day ranitidine 15 mg was added to 500 ml of water for rabbits to drink freely. The animal status was observed, and the modeling was successful 14 days after the last needle of methylprednisolone was finished.

#### Scaffold implantation procedures

All surgeries were performed by the same team of personnel under the same operating conditions. Animals were anesthetized preoperatively with ketamine and xylazine, which were administered at 40 and 4 mg/kg, respectively. Based on the intraoperative response, additional inhalation anesthesia was performed with isoflurane to maintain the depth of anesthesia.

When the animal enters deep anesthesia, a 20 mm incision is made through lateral skin, exposing the greater trochanter of femur. Core decompression surgery with a diameter of 3 mm was then performed along both hip femoral neck axes from the distal end of the greater trochanter. First, under the guidance of fluoroscopy, use a drill with a diameter of 2 mm to create a bone channel from the lateral side to the superior medial aspect of femoral head. The channel direction was at the mid shaft of femoral neck. Then use a drill bit with a diameter of 3 mm to widen the 2 mm channel. PO-PPM5 was randomly assigned and implanted into the channel of the right or left hip (as shown in Figure 1), while the other channel was not implanted with a scaffold and remained empty as a blank control. Manually press a scaffold into the bone channel.

The animals will feel pain after the anesthesia subsides. Therefore, in order to give the animals pain relief, buprenorphine should be injected two times a day in the first 2 days after the operation, with the dose of 0.5 mg/kg each time.

#### Micro-CT observation of bone repair

After 12 weeks of scaffold implantation, pentobarbital sodium was injected through ear vein. In order to kill the animals, the dose of pentobarbital sodium injection was 90–120 mg/kg. After execution, collect the samples of bilateral femurs, remove the soft tissues and put them into 10% formalin solution for fixation for 1 week. Then use micro-CT with spatial resolution of 18 μm to scan the distal femur of rabbits under the condition of voltage 66 kV and current 110 μA. In the center of the bone channel with a diameter of 3 mm, select the appropriate area and reconstruct it through the CT analyzer. Two hundred axial images were reconstructed into 3D images, and the bone volume/total volume (BV/TV), bone mineral density (BMD), bone trabecular spacing (Tb.SP), bone trabecular number (Tb.N) and bone trabecular thickness (Tb.Th) were calculated by software.

### Hematoxylin–eosin staining

Femurs were decalcified with 14% EDTA solution (pH 7.2) for 28 days. Decalcified femurs were dehydrated and embedded in paraffin for sectioning. Sections (5 μm) were stained with hematoxylin–eosin (H&E) (Servicebio). Histological sections were examined using an optical microscope (Leica, Germany).

### Statistical analysis

The experimental data were statistically analyzed with the statistical software GraphPad Prism 8. All experiments were repeated at least three times, and the results were expressed in the form of mean ± standard deviation. The paired *t*-test analysis was used to compare the two groups of data. When *P < 0.05, **P < 0.01 or ***P < 0.001, the difference was considered significant.

## Results and discussion

### Pore structure and mechanical property of PO scaffolds

**Figure 2.**
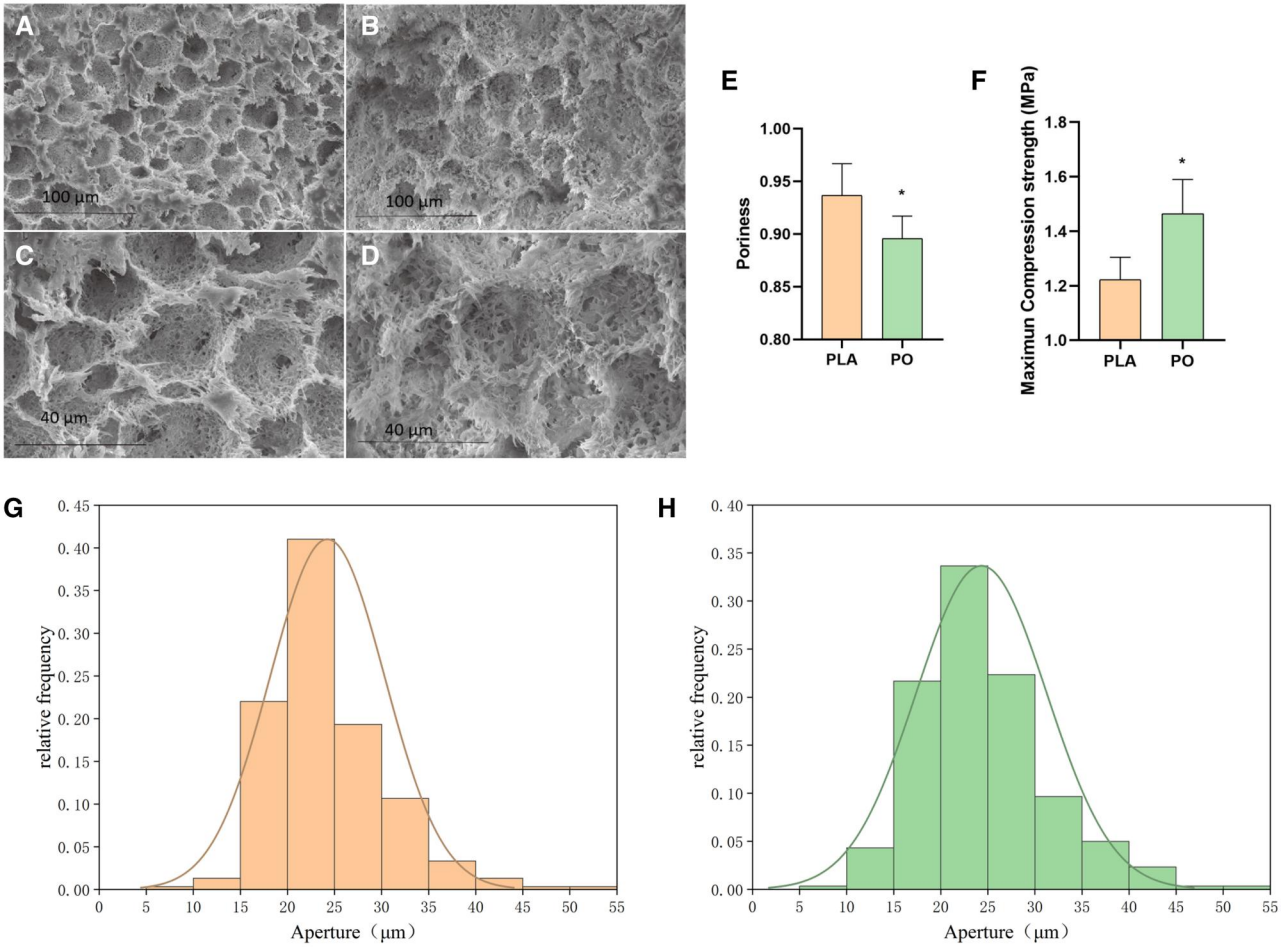
Comparison of pore size and mechanical properties of PLLA and PO scaffolds, (**A**) and (**C**) SEM images of PLLA scaffold; (**B**) and (**D**) SEM images of PO scaffold; (**E**) the pore size distribution of PLLA and PO scaffold; (**F**) the maximum compressive strength of PLLA and PO scaffold; (**G**) the pore size distribution of PLLA scaffold; (**H**) the pore size distribution of PO scaffold. Significant differences between various groups were denoted as *(P < 0.05).

Figure 2 shows the SEM images of PLLA scaffold and PO scaffold, where (**A**) and (**C**) are PLLA scaffold, (**B**) and (**D**) are PO scaffold. The PLLA and PO scaffolds prepared by phase separation method in the system of 1,4-dioxane and water are porous. Since water is well dispersed throughout the system, moisture removal by freeze dryer in the later stage of preparation enables to obtain scaffolds with more uniform pore distribution. Water has fluidity and is not disturbed by shape, so the created macropores have connected small pores between them. After loading OTF, the scaffold microtopography was not obviously affected and was still an interconnected porous structure. The pore sizes of 300 pores in the SEM images of PLLA scaffolds and PO scaffolds were counted, respectively, and their distribution was shown in the following figure.

PLLA scaffolds had an average pore size of 24.24 ± 6.09 μm with 93% of pores distributed at 15–35 μm, especially pores with pore size at 20–25 μm accounting for 41% of all pores, as shown in Figure 2G. The PO scaffolds pore size distribution situation was similar to that of PLLA scaffolds, with scaffold average pore size of 24.31 ± 6.94 μm, in which 87% of all pore diameters were distributed in 15–35 and 20–25 μm pores accounted for 34% of all pores, as shown in Figure 2H. It can be seen that loading OTF did not affect the pore structure of scaffolds. Porosity is an important indicator of scaffolds for bone repair, and according to a literature study, scaffolds with porosity >50% can achieve vascularization [27]. Figure 2E is the average of PLLA and PO scaffolds porosity. The porosity of the PLLA scaffolds was as high as 93.67%, although after loading OTF, the porosity of the scaffolds had an obvious decrease, but the porosity of the PO scaffolds still remained above 89.56%, with a statistical difference between the two (*P < 0.05). The reduction of the porosity of PO scaffold may be due to the inclusion of OTF in PLLA, which occupies the internal space of scaffold. The high porosity of PLLA and PO scaffolds meet the requirements of bone implant material porosity, facilitating the exchange of oxygen and nutrients during bone repair.

Mechanical performance is a very important index of bone implants. Different implant sites have different mechanical requirements for scaffolds. Figure 2F shows the maximum compressive strength of PLLA and PO scaffolds. According to the measurement results, both PLLA and PO scaffolds can meet the minimum strength requirements for cancellous bone repair. The mechanical properties of the scaffolds increased after adding OTF. The maximum compressive strength of the PLLA scaffolds was 1.22 MPa. After loading OTF, the strength of the scaffolds reached 1.46 MPa. The difference between the two was statistically significant (*P < 0.05). The increase in the maximum compressive strength of PO scaffolds may be due to the fact that the porosity of PO scaffolds is lower than that of PLLA scaffolds under the same substrate material.

### SEM analysis of PPM film

**Figure 3.**
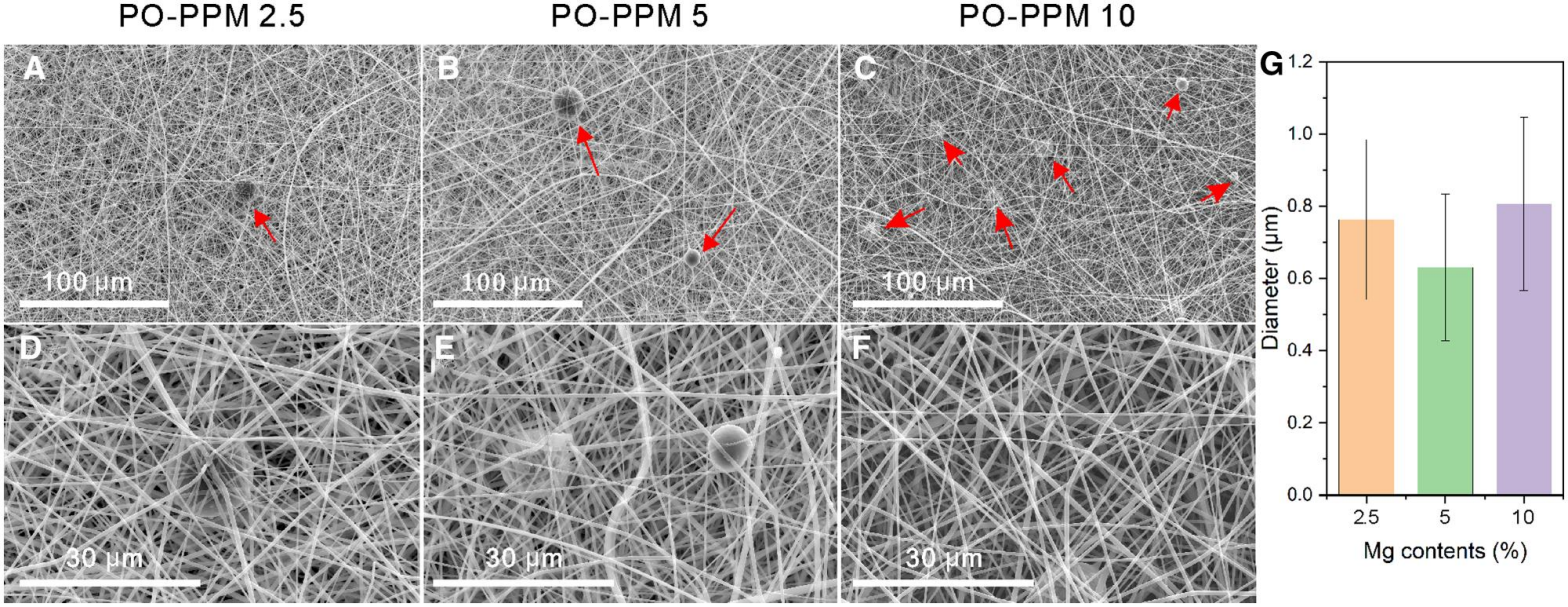
SEM of PPM scaffolds with different Mg contents, in which Mg content in (**A**) and (**D**) is 2.5%, Mg content in (**B**) and (**E**) is 5%, and Mg content in (**C**) and (**F**) is 10%; (**G**) statistical analysis of the diameter of fibers in the SEM images.

Figure 3 shows the SEM images of PPM electrospun films with different Mg content, 2.5% in (**A**) and (**D**), 5% in (**B**) and (**E**) and 10% in (**C**) and (**F**). The prepared drug-loaded electrospun film had uniform electrospinning distribution, continuous and smooth fibers, and the fiber diameter was between 300 and 900 nm. The mixed Mg particles are large, not in the fiber, but wrapped by interlaced fiber and embedded in the electrospun film, as shown by red arrow in the figure. It can be seen that with the increase of Mg content in the electrospun solution, the Mg particles in the electrospun film also increase significantly, which indicates that both low and high concentration Mg particles can be spun smoothly. The addition of magnesium particles has no significant effect on fiber diameter, as shown in Figure 3G.

### Infrared spectrum analysis of PP and PO

**Figure 4.**
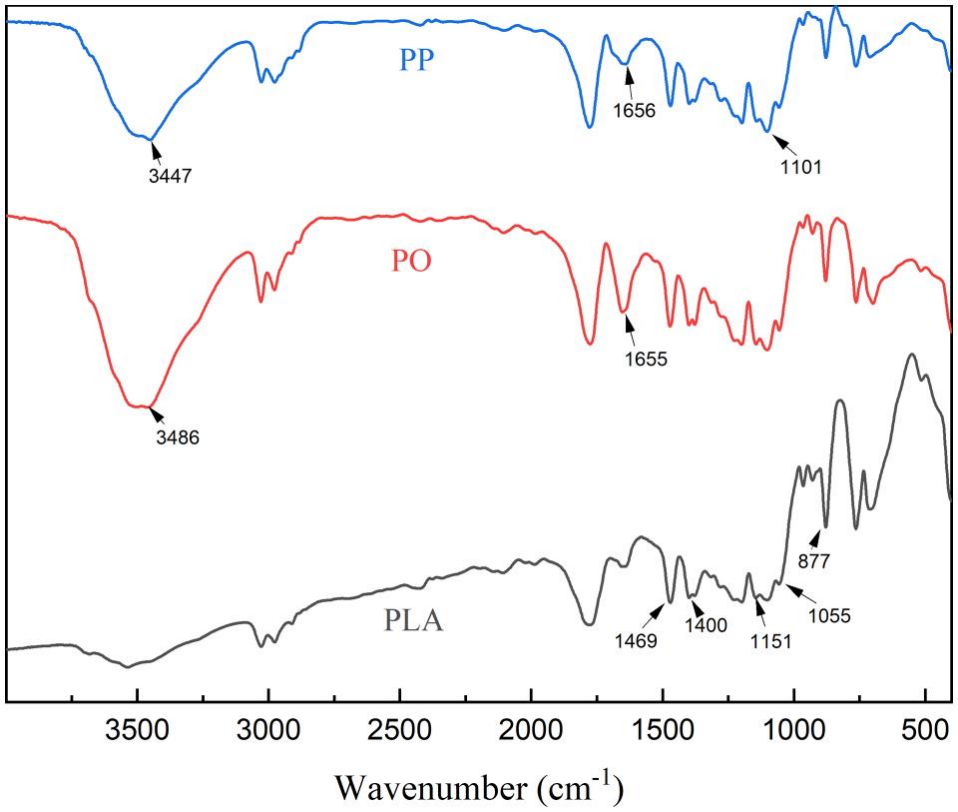
Comparison of infrared spectra of PLLA, PP and PO.

FTIR can be used to analyze the chemical structure of scaffold materials. Figure 4 contains the FTIR analysis of PO, PP and PLLA. The FTIR results of PO include not only the characteristic peak of PLLA, but also the –OH stretching vibration absorption peak with wave number of 3486 cm^−1^ and the C = O stretching vibration absorption peak in 1655 cm^−1^ aromatic ketone. These peaks are consistent with the characteristic peaks of OTF, indicating that OTF has been successfully loaded into the PO scaffold without new chemical bonds and the chemical structure of OTF itself has not been destroyed. The FTIR results of PP include not only the characteristic peak of PLLA, but also the –OH stretching vibration absorption peak with a wave number of 3447 cm^−1^, the C=C stretching vibration absorption peak with a wave number of 1656 cm^−1^, and the strong absorption peak of C–O–C stretching vibration with a wave number of 1101 cm^−1^. These peaks are consistent with the characteristic peaks of PNS, indicating that PNS has been successfully loaded into PP electrospun membrane, no new chemical bond has been generated, and the chemical structure of PNS itself has not been destroyed. Therefore, this drug loading method is feasible.

### Drug release and pH changes of PO-PPM scaffolds

**Figure 5.**
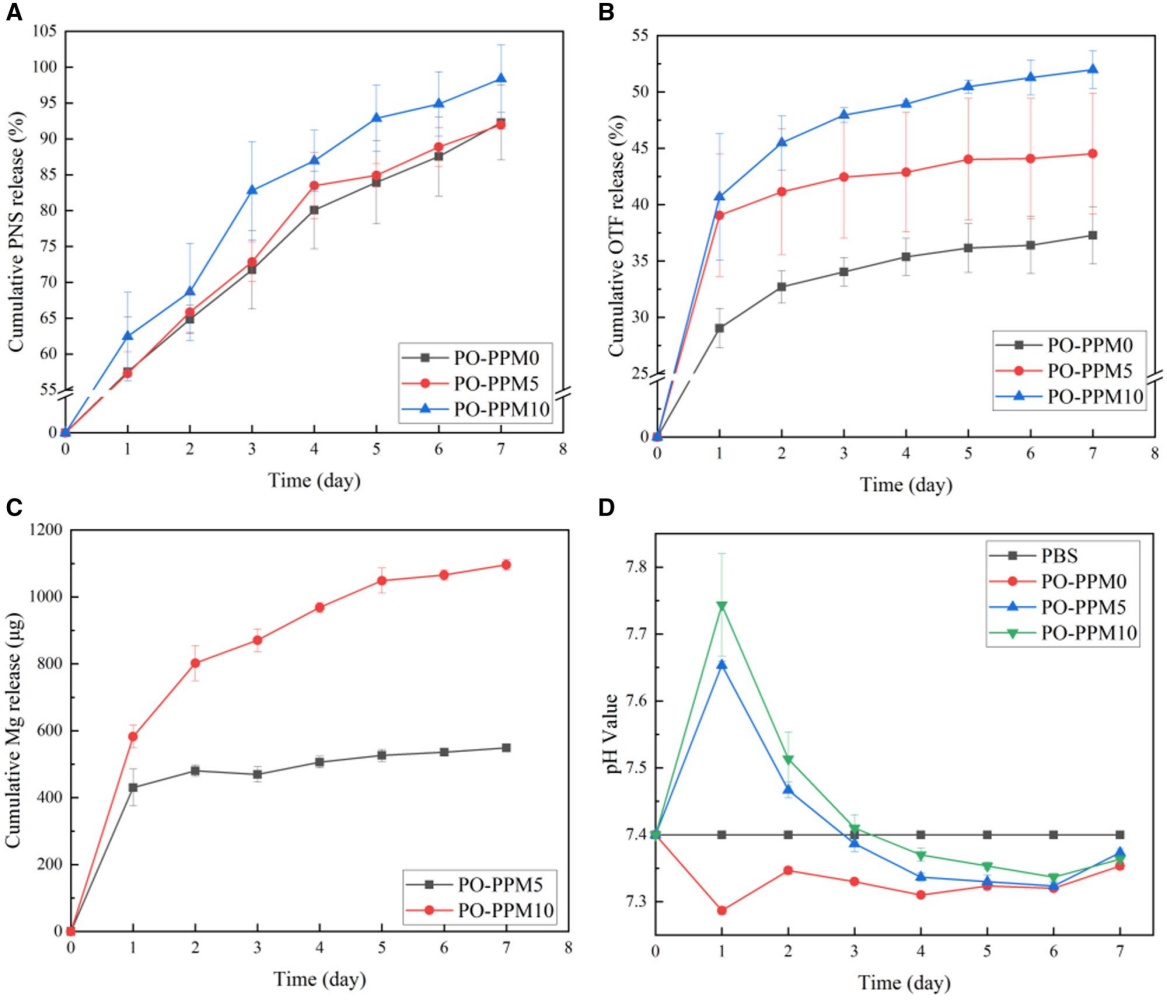
The release curve of PNS, OTF and Mg^2+^ in different Mg content scaffolds and the change of solution pH, (**A**) the release curve of PNS; (**B**) the release curve of OTF; (**C**) the release curve of Mg^2+^ and (**D**) the change of solution pH.

In the double-layer drug-loaded scaffold, OTF was located in the inner porous scaffold, PNS was located in the electrospinning of the outer electrospinning layer. And Mg, as the component that regulates drug release, existed in the gap of the electrospinning network. In the inflammatory organization stage of hematoma, the hematoma formed by the damage of periosteum and surrounding blood vessels will coagulate into blood clots within several hours, which will lead to the ischemic necrosis of some soft tissues and bone tissues, causing inflammatory reaction. PNS can relieve the hypercoagulable state of the body, inhibit the formation of local thrombus, improve microcirculation, increase vascular permeability, and accelerate the absorption and organization of hematoma. As shown in Figure 5A, the release of PNS in the three groups of scaffolds on the first day was high, reaching ∼60%. On the seventh day, the release of PNS in PO-PPM5 and PO-PPM0 reached ∼92%, and the release of PNS in PO-PPM10 scaffold reached 98%. Therefore, the scaffold can effectively exert the efficacy of PNS through its rapid release, improving the healing efficiency of hematoma inflammation at the tissue stage.

The initial callus formation stage needs 4–8 weeks. OTF can promote the proliferation and differentiation of osteoblasts, promote the expression of osteogenic related genes and effectively improve the efficiency of the initial callus formation stage. The release of OTF is shown in Figure 5B. Mg had a significant effect on the release of OTF. The release rate of the non-Mg group was the lowest. With the increase of Mg content, the release rate of OTF was significantly accelerated. On the first day, the release of OTF in the PO-PPM0 scaffold was 29%, while the release of OTF in the Mg-containing scaffold on the first day was ∼40%. Next, the release of OTF slowed down significantly. On the seventh day, the OTF released by the PO-PPM10 scaffolds was 52%, while the OTF released by the PO-PPM5 and PO-PPM0 scaffolds were 44% and 37%, respectively. Therefore, OTF can be released slowly and continuously in PBS solution to meet the demand for OTF during the initial callus formation period.

Mg can promote the release of PNS and OTF, and with the increase of Mg content, the promotion effect was more significant. This was because in the process of water intrusion from the electrospinning mesh to the inner layer, the Mg particles in the scaffold began to dissolve in water, which increased the channel of water entering the scaffold, thus promoted the release of PNS and OTF. Low concentration of Mg had no significant effect on the release of PNS, possibly due to the smaller pores formed by the dissolution of Mg at this concentration. In addition, it may be because of the large specific surface area of electrospun membranes and the PNS are easily soluble in water. With the increase of Mg particles, the pores formed by their dissolution increased, and the promotion of PNS and OTF release was more obvious.

Mg reacted with water to form Mg^2+^ and OH^–^, in which the amount of Mg^2+^ was determined by inductively coupled plasma emission spectrometry, and the pH value was directly measured by pH meter. It can be seen from Figure 5C that Mg^2+^ was continuously released throughout the release cycle. Due to the high molecular weight of PLLA used to make scaffolds, the hydrolysis within 7 days can be ignored. PO-PPM0 group did not contain Mg, so the main source of pH fluctuation was drug release. Both OTF and PNS are weakly acidic drugs. Their release was the highest on the first day, and the release was relatively slow in the next 6 days. Therefore, as shown in Figure 5D, the pH value of PO-PPM0 group reached the lowest on the first day, which is 7.28. The pH value of PO-PPM0 group rose slightly and remained relatively stable in the next 6 days, between 7.30 and 7.35. Because of the large release of Mg in the early stage of PO-PPM5 and PO-PPM10 groups, the influence of Mg on the pH value was far greater than that of OTF and PNS, so the pH value was significantly increased, and the pH value of PO-PPM10 group was higher than that of PO-PPM5 group. On the first day, the pH value of PO-PPM10 group reached 7.71, and that of PO-PPM5 group also reached 7.65. On the third day, the influence of Mg on pH was neutralized with that of OTF and PNS, so the pH value remained at ∼7.40, the same as that of PBS solution. At the later stage of release, OTF and PNS have a greater impact on the pH value, so the pH value of the solution was lower than that of PBS solution. However, due to the presence of Mg, the pH value of PO-PPM5 and PO-PPM10 groups was still higher than that of PO-PPM0 group.

### Degradation of PO-PPM scaffolds

PLLA will be hydrolyzed first *in vitro*, and the ester bonds on the main chain will be hydrolyzed to form oligomers. The hydrolysis of PLLA will be affected by the environmental pH value. In the alkaline environment, the degradation rate of PLLA was the fastest, followed by the degradation rate in the acidic environment, and the degradation rate in the neutral environment was the slowest. Because the PLLA used in this paper had a high molecular weight and a slow degradation rate, the degradation of the scaffold at 37°C for 12 weeks was observed.

**Figure 6.**
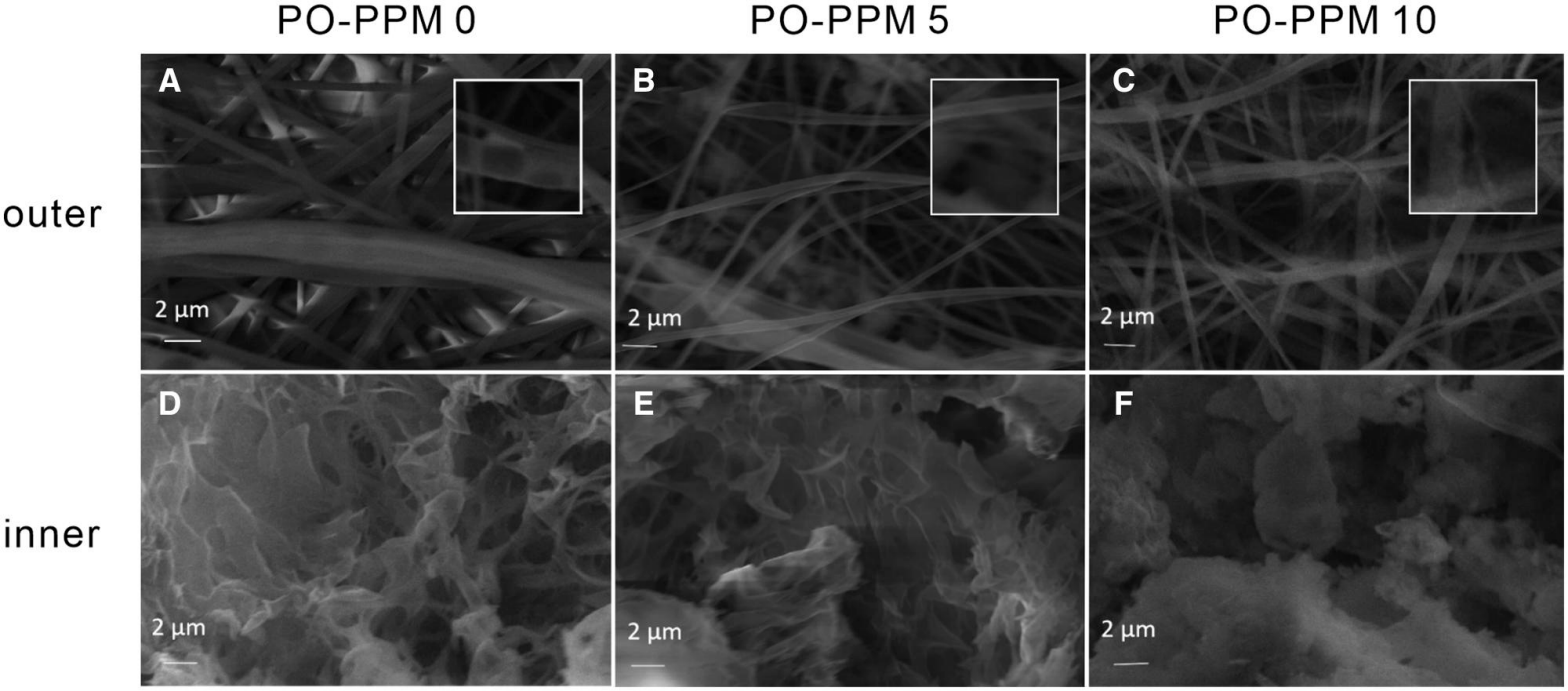
SEM images of scaffolds after 12 weeks of degradation, (**A**), (**B**) and (**C**) the outer layers of PO-PPM0, PO-PPM5 and PO-PPM10 scaffolds, respectively; (**D**), (**E**) and (**F**) the inner layers of PO-PPM0, PO-PPM5 and PO-PPM10 scaffolds, respectively, the image inside the white box is a partially enlarged image.

Figure 6 shows the micro-morphology of the scaffolds of PO-PPM0, PO-PPM5 and PO-PPM10 after 12 weeks of degradation. In order to better observe the status of scaffolds, after the scaffolds were cut along the central axis of the cylinder, the SEM images of the inner and outer layers of the scaffold were taken, respectively. SEM results showed that after 12 weeks of degradation, the electrospun fiber boundary of the outer layer of PO-PPM0 scaffold became blurred, and large holes were formed on some PLLA fibers. With the increased of Mg content, this phenomenon became more obvious, and some PLLA fibers in PO-PPM5 and PO-PPM10 scaffolds also had adhesion and fracture. However, after 12 weeks of degradation, the macropore structure in the inner layer of the scaffold remained, and the interconnected micropores between the macropores disappeared. With the increase of Mg content, the phenomenon of microporous boundary adhesion became more obvious. The results of *in vitro* degradation of the scaffold showed that the addition of Mg could promote the degradation of the scaffold.

### Animal experiment

From the test results in ‘Drug release and pH changes of PO-PPM scaffolds’ and ‘Degradation of PO-PPM scaffolds’, it is known that both PO-PPM5 and PO-PPM10 scaffolds were effective in promoting drug release and stabilizing pH value, but the pH value of PO-PPM5 group was more stable. Therefore, in order to study the ability of double-layer drug-loaded scaffold to promote bone repair, the PO-PPM5 scaffold was selected for animal experiment, and the new bone formation effect of the scaffold was analyzed by Micro-CT. In order to further study the difference of new bone formation in the bone channel, the 3D image was analyzed by software to obtain the quantitative analysis results. Quantitative analysis includes five indicators: BMD, bone volume fraction, Tb.SP, bone trabecular density and Tb.Th. BMD is an important indicator to evaluate bone strength. Bone volume fraction can reflect the change of bone mass. Trabecular separation, trabecular density and trabecular thickness are important indicators to reflect the microstructure of bone trabecular.

**Figure 7.**
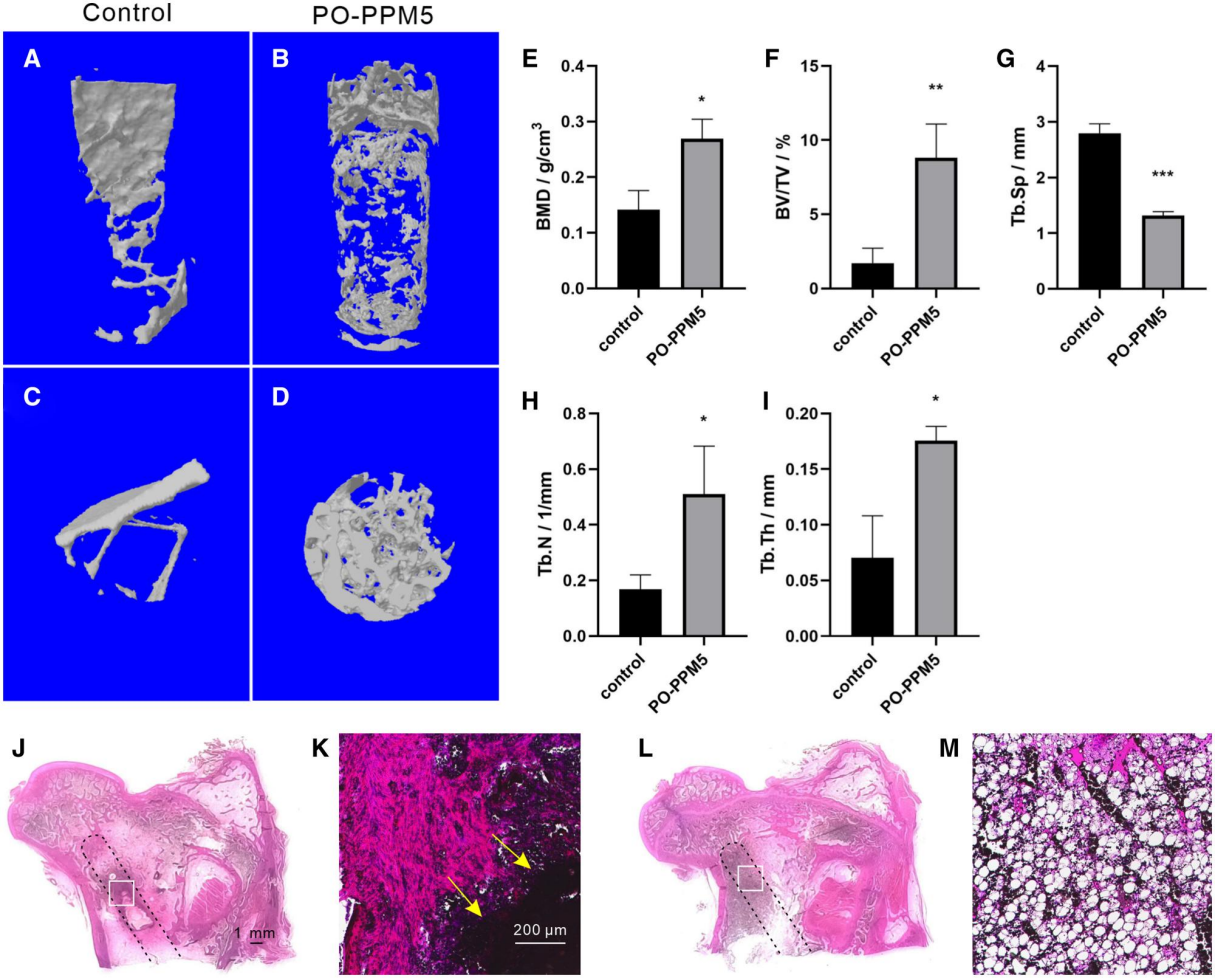
Micro-CT 3D bone reconstruction image and quantitative analysis results, in which (**A**) and (**C**) are the front and top views of the control group; (**B**) and (**D**) are the front and top views of the experimental group; (**E**), (**F**), (**G**), (**H**) and (**I**) showed BMD, BV/TV, Tb.SP, Tb.N and Tb.Th, respectively; H&E staining image of the bone channel, (**J**) and (**K**) are PO-PPM5 group, (**L**) and (**M**) are control group. Significant differences between various groups were denoted as *(P < 0.05), **(P < 0.01), or ***(P < 0.001).

Figure 7A–D shows the Micro-CT 3D bone reconstruction image in the bone channel; (**A**) and (**B**), (**C**) and (**D**) are the results of observation from the same angle. It can be clearly seen from the figure that the formation of new bone in the control group was less, and the distribution was uneven, which was thin and flaky bone. The amount of new bone formed in the PO-PPM5 group was significantly different from that in the control group, which was widely distributed and balanced, and had produced 3D bone structure. The results of quantitative analysis are shown in Figure 7E–I. The group of implanted PO-PPM5 scaffolds had higher bone density. Bone volume fraction, the density and thickness of trabeculae were also much higher than those of the control group. The trabecular separation was smaller than that of control group. The experimental results showed that the group implanted with PO-PPM5 scaffold had more bone mass, greater bone strength and better bone trabecular structure. Figure 7j–M shows the H&E staining image of the bone channel, (**J**) shows the PO-PPM5 group, (**L**) shows the control group, and the black dashed line shows the location of the bone channel. Figure 7K and M shows enlarged images of the white box areas in (**J**) and (**L**), respectively. The black area indicated by the yellow arrow in (**K**) represents the residual degradation of the scaffold. From Figure 7, it can be seen that there is significant new tissue growth around the scaffold in the PO-PPM group, while in the control group, there are numerous pores and few new tissue. Therefore, double-layer drug-loaded scaffold can effectively promote bone repair, accelerate bone repair speed and improve bone repair quality.

## Conclusion

A double-layer drug-loaded PLLA scaffold was prepared by combining the phase separation method with the electrospinning method. The inner porous PLLA scaffold was loaded with OTF, the outer electrospinning layer contained PNS in the electrospinning fibers, and the fibers contained Mg particles. The results showed that the double-layer PLLA scaffold can realize the rapid release of PNS and the continuous release of OTF, which was in line with the demand for drugs in the stage of hematoma inflammation and the stage of primary callus formation. The release rate of drugs in the scaffold can be regulated by controlling the amount of Mg particles added. Therefore, the double-layer PLLA drug-loaded scaffold had good bone repair ability.

*Conflicts of interest statement*. None declared.
